# Accuracy of duplex ultrasonography versus angiotomography for the diagnosis of extracranial internal carotid stenosis

**DOI:** 10.1590/0100-6991e-20243632-en

**Published:** 2024-05-24

**Authors:** RAUL MUFFATO DAOLIO, LUIZ FERNANDO SANTETTI ZANIN, CAROLINA DUTRA QUEIROZ FLUMIGNAN, NICOLLE CASSOLA, HENRIQUE JORGE GUEDES, JOSÉ EDUARDO MOURÃO SANTOS, JORGE EDUARDO AMORIM, LUÍS CARLOS UTA NAKANO, RONALD LUIZ GOMES FLUMIGNAN

**Affiliations:** 1 - Universidade Federal de São Paulo, Departamento de Cirurgia, Disciplina de Cirurgia Vascular e Endovascular - São Paulo - SP - Brasil; 2 - Centro Universitário São Camilo, Departamento de Cirurgia Vascular - São Paulo - SP - Brasil; 3 - Universidade Federal de São Paulo, Departamento de Diagnóstico por Imagem - São Paulo - SP - Brasil

**Keywords:** Stroke, Carotid Stenosis, Ultrasonography, Doppler, Duplex, Computed Tomography Angiography, Acidente Vascular Cerebral, Estenose das Carótidas, Ultrassonografia Doppler Dupla, Angiografia por Tomografia Computadorizada

## Abstract

**Introduction:**

Internal carotid artery (ICA) stenosis causes about 15% of ischemic strokes. Duplex ultrasonography (DUS) is the first line of investigation of ICA stenosis, but its accuracy varies in the literature and it is usual to complement the study with another more accurate exam when faced with significant stenosis. There is a lack of studies that compare DUS with angiotomography (CTA) in the present literature.

**Methods:**

we performed an accuracy study, which compared DUS to CTA of patients in a tertiary hospital with a maximum interval of three months between tests. Patients were selected retrospectively, and two independent and certified vascular surgeons evaluated each image in a masked manner. When there was discordance, a third evaluator was summoned. We evaluated the diagnostic accuracy of ICA stenosis of 50-94% and 70-94%.

**Results:**

we included 45 patients and 84 arteries after inclusion and exclusion criteria applied. For the 50-94% stenosis range, DUS accuracy was 69%, sensitivity 89%, and specificity 63%. For the 70-94% stenosis range, DUS accuracy was 84%, sensitivity 61%, and specificity 93%. There was discordance between CTA evaluators with a change from clinical to surgical management in at least 37.5% of the conflicting reports.

**Conclusion:**

DUS had an accuracy of 69% for stenoses of 50-94% and 84% for stenoses of 70-94% of the ICA. The CTA analysis depended directly on the evaluator with a change in clinical conduct in more than 37% of cases.

## RESUMO

**Introdução:**

a estenose da artéria carótida interna (ACI) causa cerca de 15% dos acidentes vasculares cerebrais isquêmicos. A ultrassonografia duplex (USD) é a primeira linha de investigação da estenose de ACI, mas sua acurácia varia na literatura e é comum complementar o estudo com outro exame de maior acurácia diante de estenose significativa. Há uma escassez de estudos que comparem a USD com a angiotomografia computadorizada (ATC) na literatura atual.

**Métodos:**

realizamos um estudo de acurácia, que comparou a USD à ATC de pacientes de um hospital terciário com um intervalo máximo de três meses entre os exames. Os pacientes foram selecionados retrospectivamente e dois cirurgiões vasculares independentes e certificados avaliaram cada imagem de maneira mascarada. Quando houve discordância, um terceiro avaliador foi convocado. Avaliou-se a precisão diagnóstica da estenose da ACI de 50-94% e 70-94%.

**Resultados:**

foram incluídos 45 pacientes e 84 artérias após a aplicação dos critérios de inclusão e exclusão. Para a faixa de estenose de 50-94%, a acurácia da USD foi 69%, sensibilidade 89% e especificidade 63%. Para a faixa de estenose de 70-94%, a acurácia da USD foi 84%, sensibilidade 61% e especificidade 93%. Ocorreu discordância entre avaliadores da ATC com mudança de conduta clínica para cirúrgica em pelo menos 37,5% dos laudos conflitantes.

**Conclusão:**

a USD teve uma acurácia de 69% para estenoses de 50-94% e de 84% para estenoses de 70-94% da ACI. A análise das ATC dependeu diretamente do avaliador com mudança de conduta clínica em mais de 37% dos casos.

## INTRODUCTION

Cerebrovascular accident (CVA) is the second most common vascular disease after acute myocardial infarction, and internal carotid artery (ICA) stenosis is associated with approximately 15% of all ischemic strokes^1^ ^-^ ^6^. Stroke is an important cause of hospitalization, morbidity, and death. The treatment of ICA stenosis is a source of intense debate, as is its clinical and radiological management, and ICA stenosis plays a central role in the practical decision for revascularization. The treatment of ICA stenosis aims to prevent cerebrovascular events and may be only clinical or involve carotid revascularization^7^ ^-^ ^9^. Surgical treatment for carotid revascularization remains indicated for stenoses of 50%-94% in symptomatic patients, that is, those who have faced a neurological event related to ICA stenosis in the last 3-6 months. The benefit is most evident in patients with stenoses above 70%. In asymptomatic patients, surgical treatment shows benefits in stenoses of 60-94%. It is essential to highlight that this 60% cut-off used the criteria from the ACAS^10^ study and that nowadays they correspond to the criteria used for stenoses above 70% according to Grant et al.^11^, which continue to be in vogue in best international practices^7^ ^,^ ^9^.

Four diagnostic tests are available for evaluating ICA stenosis: duplex ultrasonography (DUS), computerized tomography angiography (CTA) or angiotomography, magnetic resonance angiography (MRA), and digital subtraction angiography (DSA). Although DSA is the traditional standard test in this scenario, it is no longer recommended for diagnosing patients with atherosclerotic ICA stenosis, unless there are significant discrepancies in other diagnostic tests or in patients to be treated endovascularly^7^ ^-^ ^9^. DSA is invasive and associated with complications, with up to a 2% risk of stroke or death, even in exclusively diagnostic tests^9^ ^,^ ^12^ ^,^ ^13^. On the other hand, due to the high accuracy of CTA and MRA compared with DSA, CTA and MRA are also currently considered reference tests for the diagnosis of extracranial carotid stenosis^7^ ^,^ ^9^.

In this scenario, DUS has become the first choice for screening for ICA stenosis, since it is widely available, has a lower cost than the reference tests mentioned, is non-invasive, and has sufficient accuracy. The DUS technique involves the use of several ultrasound resources to determine the degree of stenosis, including B-mode morphology, color Doppler, and pulsed wave Doppler, and may also include the use of microbubble contrast^6^ ^,^ ^7^ ^,^ ^11^ ^,^ ^14^. CTA has the disadvantage of using ionizing radiation and requiring, in all cases, the administration of iodinated contrast, known to be nephrotoxic^9^. MRA does not cause exposure to ionizing radiation, but rather to a magnetic field. MRA with gadolinium contrast (paramagnetic) is more accurate than MRA without it, but it is a more expensive test, is not available in most medical centers, and is not without risks, especially in people with kidney failure in whom it can trigger nephrogenic systemic fibrosis syndrome^7^ ^,^ ^9^.

The accuracy of DUS, however, presents significant variation in the literature, depending on the criteria used in each exam, the service where it is performed, the severity of the disease examined, and the professional who performs it^7^ ^,^ ^15^. Most centers that manage the diagnosis and treatment of carotid stenosis opt for a confirmatory examination with one of the current reference tests, that is, CTA or MRA, after the initial screening with DUS and before surgical planning. In cases intended for conventional surgical treatment, this confirmation is related to the degree of stenosis and plaque characteristics^7^ ^,^ ^14^. However, in cases where endovascular treatment is indicated, CTA and MRA allow assessments essential to this revascularization technique, such as the study of the aortic arch, supra-aortic trunks, carotid bifurcation, distal ICA, and intracranial circulation^9^. When surgical treatment depends only on DUS, it is recommended that a second examination be carried out, preferably by a different evaluator^9^.

Several studies have already explored the diagnostic power of DUS, but most used it in comparison with DSA^7^. Despite the significant number of studies on the accuracy of methods to diagnose ICA stenosis, there is still lack of evidence regarding the accuracy of DUS versus MRA or CTA as reference standards, following current practice^7^. With the aim of establishing the accuracy of DUS and the degree of disagreement between examiners, we evaluated the diagnostic properties of DUS in comparison with CTA for ICA stenosis. We also assessed whether the accuracy of DUS is as reliable as to be the sole indicator for carotid revascularization.

## METHODS

We performed a retrospective study of the accuracy of DUS compared with CTA for diagnosing ICA stenosis in patients at a tertiary medical center in an upper-middle-income country. The comparison was made following the criteria of accuracy and capacity for diagnosing ICA stenosis. The local ethics committee prospectively approved the study, under registration number 75627317.0.0000.5505. Preliminary data were presented at the Society for Vascular Surgery Vascular Annual Meeting (VAM2019) and the abstract was published in the Journal of Vascular Surgery^16^. The study was also presented as preliminary data at the XVI Encontro Paulista de Cirurgia Vascular, Brazil, in 2019.

**Figure 1:**
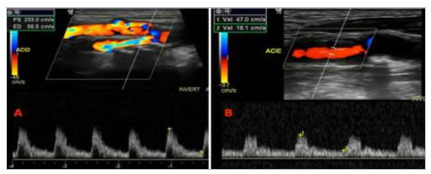
Duplex ultrasound of carotid stenosis. A: right internal carotid artery with stenosis greater than 70%. B: left internal carotid artery with stenosis less than 50% (criteria from Grant et al 2003 ^11^ ).

**Figure 2:**
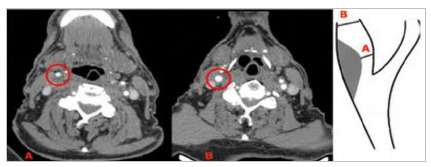
CT angiography of right internal carotid stenosis greater than 70%. A: transverse section at the point of greatest stenosis. B: transverse section at the point of normal artery distal to the stenosis (NASCET stenosis: BA/B*100 ^17^ ).

Using an electronic database registry, we searched all patients who underwent carotid DUS from January 2018 to December 2019 for possible inclusion. We excluded all patients with factors that could cause confusion during ICA analysis (e.g., arteritis, arrhythmia, and more than three months between DUS and CTA). Finally, we only included patients who underwent DUS and CTA as part of the investigation of carotid stenosis with a maximum interval of three months between exams. We used the American Consensus of Radiology to grade carotid stenosis ranges, and all unjustified definitions were based on this guideline (Table 1, Figure 1)^11^. The criteria for analyzing CTAs were based on the NASCET study (% of the stenosis diameter ratio divided by the diameter of the normal distal segment of the artery, Figure 2)^17^. We retrieved all images, and four different experienced, certified, independent examiners analyzed all images from DUS (two examiners) or CTA (two other examiners). All examiners were masked to the result of the other test and to the result of the other examiner. When both examiners in the same method disagreed on the range of stenosis, we used a third examiner as referee for that method to establish the final stenosis judgment. Data were analyzed using the Medcalc statistical software, v20.211, and reported following the STROBE guidelines^18^. Additionally, we performed an analysis of disagreement between examiners following the carotid stenosis ranges of the American Consensus of Radiology^11^. The unit of analysis for diagnostic accuracy was the ICA independently, but we used the patient as the unit of analysis for clinical outcomes such as death and stroke.

**Table 1:** American Radiology Consensus for the diagnosis of ICA stenosis with DUS.

Degree of stenosis	ICA PSV (cm /s)	Plaque estimate (%)	ICA/CCA PSV	ICA EDV (cm/s)
<50%	<125	<50	<2	<40
50-69%	125-230	≥50	<2	40-100
70-94%	>230	≥50	2-4	>100
95-99% (subocclusion)	Variable	Visible	>4	Variable
100% (occlusion)	None	Lumen not visible	Not applicable	Not applicable

*Adapted from Grant et al 2003 11. PSV: peak systolic velocity; ICA: internal carotid artery; CCA: common carotid artery; EDV: end diastolic velocity.*

## RESULTS

**Figure 3:**
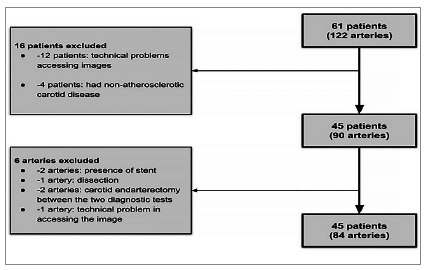
Patient flow diagram.

Of the 61 (122 ICA) patients initially selected, we excluded 12 due to technical problems accessing their images and four for having non-atherosclerotic carotid disease (e.g., vasculitis or sickle cell anemia). We also excluded two arteries due to the presence of a stent, one artery by dissection, two arteries due to a carotid endarterectomy between the two diagnostic tests of interest, and one artery due to technical problems in accessing the image. Finally, we included in our analysis 45 patients (84 arteries) who underwent DUS and CTA examinations (Figure 3). We performed independent accuracy analyzes according to ICA stenosis ranges (50%-94% and 70%-94%). We considered results positive in the range of 50%-94% stenosis, as in clinical practice this range confirms the presence of significant carotid stenoses in DUS, that is, those that produce changes in volumetric flow and velocity^19^, in addition to being the cutoff for the indication of revascularization in symptomatic patients^5^ ^,^ ^9^. A patient presenting with ICA stenosis of 50%-94% commonly undergoes a confirmatory test with a reference test (CTA or MRA) before revascularization^5^ ^,^ ^9^ ^,^ ^20^. In another analysis, we considered ICA stenosis to be positive at a 70%-94% range because it is considered more severe, associated with a greater risk of stroke and death, and this is still today the cutoff for indicating revascularization in asymptomatic patients who present additional risks^8^ ^,^ ^9^ ^,^ ^14^. The benefits of carotid revascularization in asymptomatic and symptomatic patients with 70%-94% lead us to consider this analysis crucial for clinical decision-making.

Of the 45 patients included, 37 were asymptomatic and eight were symptomatic, that is, they had previous cerebrovascular events, such as stroke, transient ischemic attack, or amaurosis fugax up to 6 months before the diagnostic examination. Most patients were neurologically symptomatic smokers with dyslipidemia and high blood pressure (Table 2).

**Table 2:** Demographic characteristics

Sex	
Men	56%
Women	44%
Age (years)	
Range	50-84
Median	67
Race	
White	68%
Brown	25%
Black	7%
Arterial hypertension	86%
Diabetes Mellitus	27%
Dyslipidemia	82%
Smokers or ex-smokers	75%
Previous myocardial infarction	14%
Previous stroke, TIA, or amaurosis fugax	52%

CVA: cerebrovascular accident; TIA: transient ischemic attack

### Carotid artery stenosis of 50% to 94%

The prevalence of ICA stenosis of 50%-94% was 21.4%. We found an accuracy of 69%, sensitivity of 89%, and specificity of 63%, in addition to a positive predictive value of 44% and a negative predictive value of 96%.

### Carotid artery stenosis of 70% to 94%

The prevalence of ICA stenosis of 70%-94% was 17.8%. In this range, we found an accuracy of 84%, sensitivity of 61%, and specificity of 93%, in addition to a positive predictive value of 73% and a negative predictive value of 87%.

**Figure 4:**
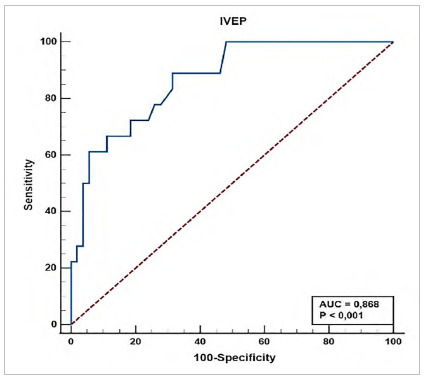
ROC curve of the 70%-94% stenosis range. AUC: area under the curve; IVEP: visual dependence index of postural stability.

The ROC curve of this stenosis range is shown in Figure 4.

### Variations between examiners

**Figure 5:**
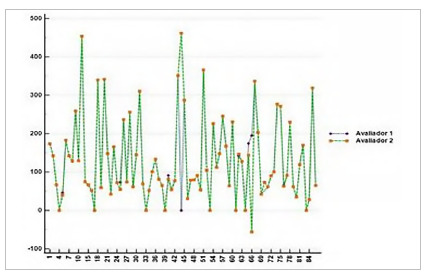
Dispersion between duplex ultrasound examiners. The X axis shows the number of arteries and the Y axis, the peak systolic velocity (PSV) in cm/s.

DUS examiners disagreed on 3% of their assessments. Figure 5 shows the dispersion between the evaluation of examiners 1 and 2, the X axis being the number of arteries and the Y axis being the systolic peak velocity (SPV).

CTA examiners, on the other hand, using this same analysis parameter, displayed a variation of 14%. We performed a correlation analysis using the Pearson test in three different scenarios. In these three analyses, we used only the 24 arteries identified as discordant in the CTA evaluator’s assessment. Table 4 describes the three scenarios. Examiner 1 versus Examiner 2: Correlation coefficient r: 0.5993, p-value: 0.020, 95% confidence interval for r (95% CI): 0.2583-08075. Examiner 1 versus referee: Correlation coefficient r: 0.6102, p-value: 0.020, r 95% CI: 0.2744-0.8134. Examiner 2 versus referee: Correlation coefficient r: 0.5369, p-value: 0.0015, r 95% CI: 0.1705-0.7729.

[Table t3]

**Table 3 t3:** Accuracy of DUS for diagnosing stenosis.

Stenosis >50%			Stenosis from 70% to 94%		
	CTA >50%	CTA <50%		CTA >70%	CTA <70%
DUS > 50%	16	20	DUS > 70%	11	4
DUS < 50%	2	34	DUS < 70%	7	50

**Table 4:** Variation between CTA examiners..

	Examiner 1 versus Examiner 2	Examiner 1 versus Referee	Examiner 2 versus Referee
Correlation coefficient r	0.5993	0.6102	0.5369
p-value	0.020	0.0015	0.0068
r (95% CI)	0.2583 - 0.8075	0.2744 - 0.8134	0.1705 - 0.7729

**Figure 6:**
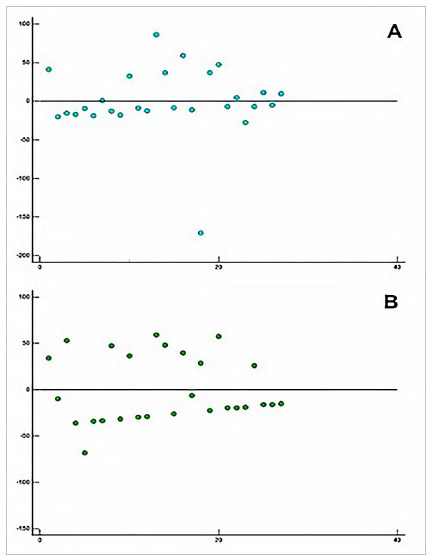
Dispersion between CTA examiners, with the points of each artery with discordance, the X axis being the number of arteries and the Y axis being the stenosis value. A: distribution of arteries with disagreement - Examiner 1; B: distribution of arteries with disagreement - Examiner 2.

Figure 6 shows the dispersion between CTA evaluators, with the points of each artery with discordance, the X axis being the number of arteries and the Y axis being the stenosis value.

The analysis of variation in CTA evaluations shows that in 37.5% of discordant arteries (9/24), treatment could be significantly altered depending on which report to be followed, varying from approach to revascularization. Table 5 illustrates the conduct highlighted by examiner.

**Table 5:** Change in conduct of CTA Examiners.

Examiner 1		Examiner 2	
Surgical	Non-Surgical	Surgical	Non-Surgical
1	8	1	13

## DISCUSSION

DUS is generally considered an examiner-dependent diagnostic test because there is variability during image acquisition. Furthermore, thresholds for defining the reach of the carotid arteries may vary between laboratories. A study evaluating the criteria used in different American centers^21^ identified that, despite attempts at standardization, only 46% of centers used the criteria determined in the American Consensus of Radiology^11^ ^,^ ^20^ ^-^ ^23^. Furthermore, there is a need for centers to audit their results, as the other studies were carried out with different professionals and ultrasound devices and do not necessarily represent the reality of accuracy in all centers^11^. In this scenario, this study addresses the lack of evidence comparing DUS with CTA for the diagnosis of ICA stenosis, as pointed out in a recent systematic review^7^, and was designed to determine the diagnostic power of DUS in our tertiary center. Our primary objective was to determine the accuracy and value of our center’s sensitivity and specificity based on data from our patients under investigation for carotid stenosis (symptomatic or asymptomatic). For the 50%-94% stenosis range, we found a sensitivity of 93% and specificity of 53%, which reveals many false positive cases in our sample. The overall accuracy of 70% was similar to other international studies^7^ ^,^ ^24^. For the stenosis range of 70%-94%, we found an overall accuracy of 84%, sensitivity of 61%, and specificity of 93%, similar to previous research^7^ ^,^ ^24^ ^,^ ^25^.

Although our data are similar in terms of accuracy values to other previous primary and secondary studies, we must highlight some relevant differences. Cassola et al.^7^ performed a Cochrane systematic review in 2022 to compare DUS with the three current reference tests (CTA, MRA, and DSA) in symptomatic patients. They included 22 studies (4,957 arteries), but only two (685 arteries) compared DUS with CTA. A broad search was carried out in at least nine different databases with electronic search strategies with no limit on language or year of publication, analyzing the risk of bias according to QUADAS-2^26^. They concluded that, for the comparison of DUS and CTA, sensitivity ranged from 57% to 94%, with specificity between 87% and 98%, where a meta-analysis was infeasible due to lack of good quality data, especially for the stenosis range of 50%-69%.

Zavanone et al^24^ proposed a systematic review in 2011 to compare the results of DUS and CTA but carried out an electronic search in a single database (PubMed), limiting the searches to the year of publication (2000-2009), using only two keywords, without providing a complete search strategy that allows reproducibility or verification of its results. They included four studies (431 arteries), without identifying symptomatic or asymptomatic patients, observing an overall accuracy of 78% and a 14%-17% disagreement between methods.

In 2019, Rustempasic et al.^27^ analyzed data from 42 of the 297 possible patients over a four-year inclusion period and used the NASCET^17^ criteria for both exams (DUS and CTA). In other words, they included a stenosis range of 30%-69%, which is unusual and no longer practiced in ultrasound services around the world since the 2003 consensus^11^. They only concluded that DUS correlates positively with CTA, without providing details of sensitivity, specificity, or accuracy.

In 2018, Birmpili et al.^25^ retrospectively analyzed images of 100 arteries to evaluate the accuracy of CTA using DUS as a reference test. It is a peculiar study, as CTA is usually used as a reference and not the other way around, since DUS would be the least invasive screening test. This study did not distinguish between symptomatic or asymptomatic patients and found an agreement lower than usual between manual methods (Kappa 0.34).

The secondary objective of our study was to evaluate whether our overall accuracy allows us to responsibly indicate carotid endarterectomy without data from another imaging test with greater accuracy (reference test), such as CTA or MRA. In our service, we use CTA in most cases. With our results, in agreement with the data already shown in a recent systematic review^7^, DUS is accurate in discriminating the presence or absence of significant stenosis of the carotid artery (< 50% or 50%-94%), but with a considerable rate of false positives and high sensitivity, being a good test to rule out ICA stenosis, but still requiring another DUS test to confirm a positive result. We therefore conclude that this second DUS exam is necessary to confirm the diagnosis in patients with a positive DUS result, corroborating international data^9^.

We also analyzed the disagreement between DUS and CTA examiners. We noticed a higher variation between CTA examiners (14%) when compared with DUS ones (3%), which denotes highly relevant information, given that DUS is a traditionally evaluator-dependent exam. We chose to perform a dispersion test only for CTA because it is the most accurate test, whose data determine clinical management, and because the divergence from DUS was minimal. In our analysis, this variation may be relevant to the point where the patient is referred for clinical or surgical treatment depending on their CTA evaluators. Similar data have been described in previous studies^28^. However, our study was more decisive in this conclusion, since the variation leads to a relevant change in behavior that could not be ignored.

As limiting factors of our study, many patients did not meet the minimum inclusion criteria because they did not undergo both exams within a minimum period of three months or in the same symptomatic situation (they had some neurological event between the exams). The lack of financial resources in the Brazilian public health system, Sistema Único de Saúde (SUS), may be related, assuming that some necessary exams are delayed, or higher-cost technologies are not available at specific scenarios. Another limitation is the retrospective design of this study, which predispose to incomplete data in patient records and difficulty in accessing exams and clinical follow-up information. The fact that all exams were performed in a clinical environment may have contributed to bias, as sonographers could recognize a result, consult the patient’s clinical data, and know which patient is most likely to have a significant stenosis. Finally, disagreement between CTA examiners may have affected DUS accuracy, even with our efforts to reduce this bias with two masked examiners and the possibility of a referee review.

As a strong point of our study, we highlight our efforts to reduce bias by having two masked evaluators for each exam, the possibility of a referee, and the standardized evaluation, following international criteria established for each test used, that is, DUS and CTA^11^ ^,^ ^17^. It is an important study in a scenario of scarcity of similar evidence, especially for the stenosis range of 50%-69%, and high variability of DUS accuracy between centers around the world, opening the possibility of including good quality systematic reviews and meta-analyses it in future^7^ ^,^ ^24^ ^,^ ^25^ ^,^ ^27^.

## CONCLUSION

In the stenosis range of 50%-94%, the accuracy of DUS was 69%, the sensitivity was 89%, and the specificity was 63%. In the 70%-94% stenosis range, the accuracy of DUS was 84%, the sensitivity was 61%, and the specificity was 93%. CTA examiners (14%) varied more in their assessment of stenosis than DUS ones (3%), enabling changes in clinical decision making. Diagnostic accuracy tests are not fixed properties, they describe the behavior of a test under specific conditions, and our data are crucial in determining the real diagnostic power of DUS compared with CTA.

## References

[1] Cortesi PA, Fornari C, Madotto F (2021). Trends in cardiovascular diseases burden and vascular risk factors in Italy The Global Burden of Disease study 1990-2017. Eur J Prev Cardiol.

[2] Brant LCC, Nascimento BR, Veloso GA (2022). Burden of Cardiovascular diseases attributable to risk factors in Brazil data from the "Global Burden of Disease 2019" study. Rev Soc Bras Med Trop.

[3] Roth GA, Mensah GA, Johnson CO (2019). Global Burden of Cardiovascular Diseases and Risk. Factors, 1990-.

[4] Avan A, Digaleh H, Di Napoli M (2019). Socioeconomic status and stroke incidence, prevalence, mortality, and worldwide burden an ecological analysis from the Global Burden of Disease Study 2017. BMC Med.

[5] Flumignan CDQ, Flumignan RLG, Navarro TP (2017). Estenose de carótida extracraniana revisão baseada em evidências. Rev. Col. Bras. Cir.

[6] Flumignan CDQ, Flumignan RLG, Nakano LCU (2017). Spontaneous carotid dissection. Rev Assoc Med Bras.

[7] Cassola N, Baptista-Silva JC, Nakano LC (2022). Duplex ultrasound for diagnosing symptomatic carotid stenosis in the extracranial segments. Cochrane Database Syst Rev.

[8] Clezar CN, Cassola N, Flumignan CD (2023). Pharmacological interventions for asymptomatic carotid stenosis. Cochrane Database Syst Rev.

[9] Naylor R, Rantner B, Ancetti S (2023). Editor's Choice - European Society for Vascular Surgery (ESVS) 2023 Clinical Practice Guidelines on the Management of Atherosclerotic Carotid and Vertebral Artery Disease. Eur J Vasc Endovasc Surg.

[10] Walker MD, Marler JR, Goldstein M (1995). Endarterectomy for Asymptomatic Carotid Artery Stenosis. JAMA.

[11] Grant EG, Benson CB, Moneta GL (2003). Carotid artery stenosis gray-scale and Doppler US diagnosis--Society of Radiologists in Ultrasound Consensus Conference. Radiology.

[12] Davies KN, Humphrey PR (1993). Complications of cerebral angiography in patients with symptomatic carotid territory ischaemia screened by carotid ultrasound. J Neurol Neurosurg Psychiatry.

[13] Dawkins AA, Evans AL, Wattam J (2007). Complications of cerebral angiography a prospective analysis of 2,924 consecutive procedures. Neuroradiology.

[14] Geiger MA, Flumignan RLG, Sobreira ML (2022). Carotid Plaque Composition and the Importance of Non-Invasive in Imaging Stroke Prevention. Front Cardiovasc Med.

[15] Curley PJ, Norrie L, Nicholson A (1998). Accuracy of carotid duplex is laboratory specific and must be determined by internal audit. Eur J Vasc Endovasc Surg.

[16] Daolio RM, Cassola N, Flumignan C (2019). PC126 Accuracy of Vascular Ultrasound Compared With Computed Tomography Angiography for Extracranial Carotid Stenosis Imaging. J Vasc Surg.

[17] NASCET Collaborators (1991). Beneficial effect of carotid endarterectomy in symptomatic patients with high-grade carotid stenosis North American Symptomatic Carotid Endarterectomy Trial Collaborators. N Engl J Med.

[18] von Elm E, Altman DG, Egger M (2008). The Strengthening the Reporting of Observational Studies in Epidemiology (STROBE) statement guidelines for reporting observational studies. J Clin Epidemiol.

[19] Spencer MP, Reid JM (1979). Quantitation of carotid stenosis with continuous-wave (CW) Doppler ultrasound. Stroke.

[20] Brott TG, Halperin JL, Abbara S (2013). 2011 ASA/ACCF/AHA/AANN/AANS/ACR/ASNR/CNS/SAIP/SCAI/SIR/SNIS/SVM/SVS guideline on the management of patients with extracranial carotid and vertebral artery disease executive summary: a report of the American College of Cardiology Foundation/American Heart Association Task Force on Practice Guidelines, and the American Stroke Association, American Association of Neuroscience Nurses, American Association of Neurological Surgeons, American College of Radiology, American Society of Neuroradiology, Congress of Neurological Surgeons, Society of Atherosclerosis Imaging and Prevention, Society for Cardiovascular Angiography and Interventions, Society of Interventional Radiology, Society of NeuroInterventional Surgery, Society for Vascular Medicine, and Society for Vascular Surgery. Developed in collaboration with the American Academy of Neurology and Society of Cardiovascular Computed Tomography. Catheter Cardiovasc Interv.

[21] Columbo JA, Suckow BD, Griffin CL (2017). Carotid endarterectomy should not be based on consensus statement duplex velocity criteria. J Vasc Surg.

[22] Kim AH, Augustin G, Shevitz A (2018). Carotid Consensus Panel duplex criteria can replace modified University of Washington criteria without affecting accuracy. Vasc Med.

[23] Barlinn K, Floegel T, Kitzler HH (2016). Multi-parametric ultrasound criteria for internal carotid artery disease-comparison with CT angiography. Neuroradiology.

[24] Zavanone C, Ragone E, Samson Y (2012). Concordance rates of Doppler ultrasound and CT angiography in the grading of carotid artery stenosis a systematic literature review. J Neurol.

[25] Birmpili P, Porter L, Shaikh U, Torella F (2018). Comparison of Measurement and Grading of Carotid Stenosis with Computed Tomography Angiography and Doppler Ultrasound. Ann Vasc Surg.

[26] Whiting PF (2011). QUADAS-2 A Revised Tool for the Quality Assessment of Diagnostic Accuracy Studies. Ann Intern Med.

[27] Rustempasic N, Gengo M (2019). Assesment of Carotid Stenosis with CT Angiography and Color Doppler Ultrasonography. Med Arch.

[28] Patel SG (2002). Outcome, observer reliability, and patient preferences if CTA, MRA, or Doppler ultrasound were used, individually or together, instead of digital subtraction angiography before carotid endarterectomy. J Neurol Neurosurg Psychiatry.

